# A direct interaction between fascin and microtubules contributes to adhesion dynamics and cell migration

**DOI:** 10.1242/jcs.175760

**Published:** 2015-12-15

**Authors:** Giulia Villari, Asier Jayo, Jennifer Zanet, Briana Fitch, Bryan Serrels, Margaret Frame, Brian M. Stramer, Benjamin T. Goult, Maddy Parsons

**Affiliations:** 1 Randall Division of Cell and Molecular Biophysics, King's College London, Guys Campus, London SE1 1UL, UK; 2 Université de Toulouse, Université Paul Sabatier and Centre National de la Recherche Scientifique, Unité Mixte de Recherche 5547, Centre de Biologie du Développement, Toulouse F-31062, France; 3 Edinburgh Cancer Research UK Centre, Institute of Genetics and Molecular Medicine, University of Edinburgh, Western General Hospital, Edinburgh EH4 2XR, UK; 4 School of Biosciences, University of Kent, Canterbury CT2 7NJ, UK

**Keywords:** Fascin, Cytoskeleton, Actin, Microtubule, Focal adhesion, Migration, Focal adhesion kinase

## Abstract

Fascin is an actin-binding and bundling protein that is highly upregulated in most epithelial cancers. Fascin promotes cell migration and adhesion dynamics *in vitro* and tumour cell metastasis *in vivo*. However, potential non-actin bundling roles for fascin remain unknown. Here, we show for the first time that fascin can directly interact with the microtubule cytoskeleton and that this does not depend upon fascin-actin bundling. Microtubule binding contributes to fascin-dependent control of focal adhesion dynamics and cell migration speed. We also show that fascin forms a complex with focal adhesion kinase (FAK, also known as PTK2) and Src, and that this signalling pathway lies downstream of fascin–microtubule association in the control of adhesion stability. These findings shed light on new non actin-dependent roles for fascin and might have implications for the design of therapies to target fascin in metastatic disease.

## Abstract (precis)

**Summary:** Fascin associates directly with microtubules independently of F-actin binding, and this contributes to microtubule dynamics, adhesion assembly and cell migration.

## INTRODUCTION

Fascin is a highly conserved, 55-kDa actin-binding and bundling protein that is required for the maintenance and stability of parallel bundles of filamentous (F-) actin in a variety of cellular contexts (Jayo and Parsons, 2010). Fascin is highly upregulated in most invasive cancers and is increasingly recognized as a prognostic marker of metastatic disease and has thus received considerable recent attention as a potential therapeutic target (Bi et al., 2012; Gao et al., 2012; Hashimoto et al., 2006; Jayo and Parsons, 2010; Li et al., 2008). Actin bundling occurs through two sites in fascin at the N- and C-terminus of the protein. The best characterised of these sites lies between amino acids 33 and 47, through which actin binding is negatively controlled by protein kinase C (PKC)-dependent phosphorylation on S39. Modification of this site has also been shown to play a role in association between fascin and p75NTR (also known as NGFR), Rab35 and LIM kinase (LIMK1), the latter of which supports fascin-dependent filopodia stabilisation (Jayo et al., 2012; Shonukan et al., 2003; Zhang et al., 2009). Previous structural studies have proposed a second actin-binding domain between amino acids 277 and 493, but how this site is regulated is not yet understood (Ono et al., 1997).

Fascin–actin binding has been shown to play a central role in the regulation of adhesion, migration and invasion in a range of cell types and contexts (Jayo and Parsons, 2010). Most of these functions have been attributed to fascin-dependent effects on filopodia stabilisation that ultimately impact upon efficient directed migration and invasion. We have previously shown that depletion of fascin results in significantly reduced focal adhesion dynamics (Hashimoto et al., 2007) and recent work has shown this partially requires the canonical actin-bundling function of fascin to modulate cell contractility (Elkhatib et al., 2014). Dynamic focal adhesions are known to be under the control of both actin and microtubule (MT) cytoskeletons; F-actin stress fibres provide an anchor for focal adhesion stability and growth, whereas MTs have been shown to target adhesion sites and mediate disassembly (Ciobanasu et al., 2013; Etienne-Manneville, 2013). We have recently shown that phosphorylation of S274 (S289 in *Drosophila*) within the second actin-binding site also plays a key role in controlling fascin–actin bundling and filopodia assembly *in vitro* and *in vivo* (Zanet et al., 2012). However, fascin mutated at S274 is able to support cell motility independently of actin bundling, suggesting that other, currently unknown, binding partners or functions exist for this molecule that contribute to cell adhesion and migration (Zanet et al., 2012).

In the present study, we set out to test the hypothesis that fascin has non-actin-bundling-dependent binding partners that contribute to cell adhesion and migration. Our results provide evidence of a new interaction between fascin and the MT cytoskeleton, and show that this association does not require fascin–actin bundling. Loss of fascin results in more stable MTs in cells and *in vivo*, and blocking fascin–MT binding leads to more stable adhesions and slower cell migration. We further show that dynamic MTs promote the formation of a complex between fascin, focal adhesion kinase (FAK, also known as PTK2) and Src. Expression of MT-binding mutants of fascin leads to more stable adhesions, and this phenotype can be reversed by overexpressing constitutively active Src, contributing to focal adhesion disassembly. Thus our data show a novel role for fascin in control of migration through associating with MTs. This has broader implications for understanding fascin in other biological contexts as well as in the design of therapeutic agents to inhibit metastatic disease.

## RESULTS

### Depletion of fascin results in larger focal adhesions and less-dynamic MTs

**Fig. 1.**
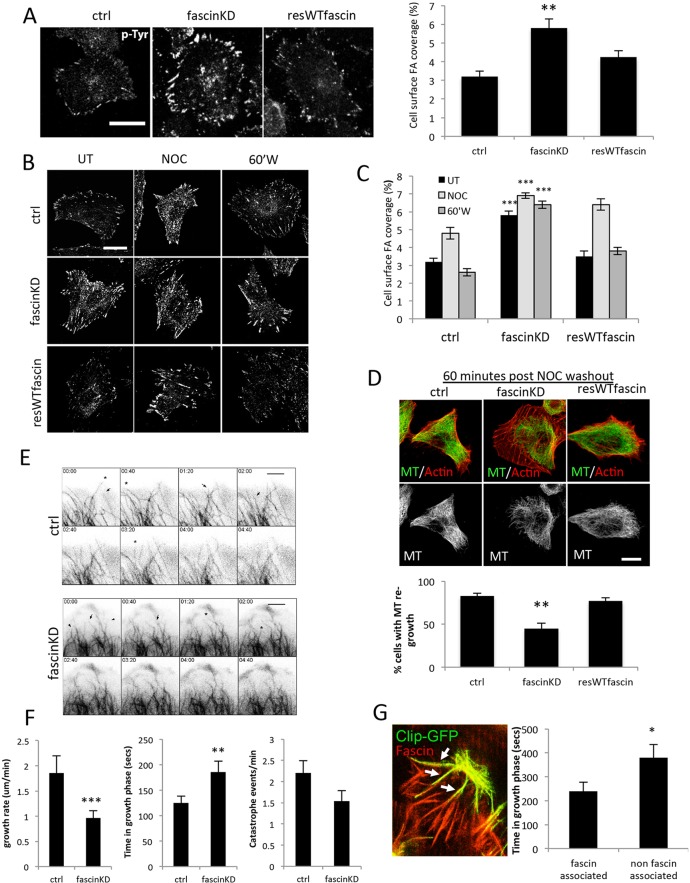
**Fascin regulates focal adhesion size and MT dynamics.** (A) Representative images of phosphotyrosine (p-Tyr)-stained MDA MB 231 cells expressing control (ctrl) shRNA, fascinKD shRNA or fascinKD shRNA and WT fascin–GFP (resWTfascin). Scale bar: 20 μm. The bar graph shows the mean±s.e.m. quantification of the percentage focal adhesion coverage area per cell calculated from 50 cells per condition over three independent experiments. (B) Representative images of MDA MB 231 cells as in A, that was either untreated (UT), treated with NOC for 20 min (NOC) or at 60 min post-NOC washout (60′W). Scale bar: 20 μm. (C) A bar graph representing the mean±s.e.m. quantification of the percentage of the cell surface covered by focal adhesions from images similar to those in B. *n*=40 cells per condition over three independent experiments. (D) Example images of control or fascinKD cells stained for F-actin (red) or tubulin (green) at 60 min after NOC washout. The graph below shows mean±s.e.m. values of MT re-growth from *n*=30 cells per experiment. Images are representative of findings across three independent experiments. (E) Example images taken from time-lapse confocal microscopy movies of control or fascinKD HeLa cells expressing tubulin–mCherry. Full movies are shown in Movie 1. Arrows and arrowheads denote growing or stable MTs, respectively. Asterisks denote catastrophe events. (F) Bar graphs showing mean±s.e.m. quantification of MT growth rate, time spent in growth phase and MT catastrophe events per minute calculated from 15 MTs per cell in six cells per experiment over four independent experiments. (G) Representative image of live *Drosophila* haemocytes within living embryos co-expressing mCherry–fascin and Clip–GFP. Arrows indicate regions of colocalisation between fascin and Clip. The graph shows quantification from movies of live *Drosophila* haemocytes within living embryos co-expressing mCherry–fascin and Clip–GFP. Growth of fascin-associated and non-associated MTs are shown. Values are pooled from 95 MTs analysed from eight cells across six independent movies. An example time-lapse is shown in Movie 2. Bars show mean±s.e.m. *P<0.05; **P<0.01; ***P<0.001 compared to controls (A,C,D, Students *t*-test; F,G, one-way ANOVA). Scale bars: 20 μm.

To analyse the role of fascin in controlling focal adhesion assembly, we generated MDA MB 231 and HeLa human cancer cell lines stably expressing short hairpin RNA (shRNA) to efficiently deplete endogenous fascin (fascinKD) followed by expression of wild-type fascin–GFP (resWT; Fig. S1A). Analysis of fixed cells stained for phosphorylated tyrosine (p-Tyr) as a focal adhesion marker demonstrated that knockdown of fascin increased the percentage of MDA MB 231 cell area occupied by focal adhesions compared to control or resWT cells (Fig. 1A), but with no change in total adhesion protein levels (Fig. S1B) or spread cell area (data not shown). We also saw similar changes in focal adhesion coverage in HeLa cells depleted of fascin (Fig. S1C), which is consistent with our own previous observations (Hashimoto et al., 2007) and a more recent report in colon carcinoma cells (Elkhatib et al., 2014). This finding was further confirmed in mouse NIH3T3 fibroblasts, which also express high levels of endogenous fascin, suggesting that this role for fascin also extends to non-tumour cells (Fig. S1D). To enable quantitative analysis of focal adhesion disassembly in a synchronised manner, we performed nocodazole (NOC) washout assays in control, fascinKD and resWT cells. NOC induces MT depolymerisation and treatment of cells with this drug has previously been shown to result in focal adhesion growth followed by rapid adhesion disassembly upon drug washout (Ezratty et al., 2005). Analysis of adhesions in fascin-depleted or rescued MDA MB 231 cells demonstrated a significant reduction in dynamic focal adhesion growth and disassembly upon NOC treatment and fascin knockdown (Fig. 1B,C). We further confirmed this defective focal adhesion disassembly upon MT disruption in fixed HeLa and NIH 3T3 cells (Fig. S1C,D). Live-cell time-lapse confocal imaging also demonstrated a significantly reduced adhesion disassembly response to NOC washout in fascin-depleted MDA MB 231 cells expressing vinculin–mRFP (Fig. S1E). These data combined suggest that fascin is required for regulated MT-dependent focal adhesion dynamics in both tumour-derived and non-cancer cell types.

To determine whether loss of fascin had an impact on MT growth, we fixed control, fascin-knockdown and resWT cells throughout the NOC washout assay and stained these cells for tubulin. Images of these cells revealed that loss of fascin led to a significant delay in MT re-growth following NOC washout as compared to control cells (Fig. 1D). F-actin staining also demonstrated that fascinKD cells retained thicker stress fibres at 60 min after washout in agreement with previous observations in fascin-depleted colon carcinoma cells (Elkhatib et al., 2014). To investigate whether the observed defects in fascin-silenced cells were due to effects on MT dynamics, we performed time-lapse imaging of tubulin–mCherry-expressing control or fascinKD HeLa cells under basal conditions. HeLa cells represent an excellent and well characterised model cell line to study MT dynamics as they have a large cytoplasmic spread surface area to study individual MTs by fluorescence time-lapse microscopy. Analysis of individual MT behaviour over time (Fig. 1E for snapshots; Movie 1) revealed that depleting fascin resulted in MTs becoming significantly more stable, with exhibiting slower growth and fewer catastrophe events compared to MTs in control shRNA cells (quantification in Fig. 1F) in agreement with the analysis of MT re-growth following NOC washout. Western blotting further demonstrated that acetylated tubulin, a marker of stable MTs (Webster and Borisy, 1989), was increased in fascin-silenced cells (Fig. S2A). These data combined demonstrate that fascin contributes to the dynamics and stability of MTs.

We next wanted to determine whether fascin plays a role in MT stability *in vivo*. We have previously shown that fascin is required for cell migration in haemocyte blood cells within the developing *Drosophila* embryo, where it can stabilise F-actin bundles at the leading edge (Zanet et al., 2012, 2009). In order to determine whether fascin could also co-operate with MT within an *in vivo* setting, we imaged fascin–mCherry and the MT-binding domain of human Clip170 tagged to GFP [CLIP–GFP; a plus-end-tracking protein (+TIP)] in migrating haemocytes in developing *Drosophila* embryos. Images demonstrated that partial colocalisation between fascin and MT bundles occurred within the lamellae, and these MT bundles have previously been shown to be required for directed migration in these cells (Fig. 1G; Stramer et al., 2010). As our data in human and mouse cells demonstrated a role for fascin in promoting dynamic MTs, we analysed the growth phase time of MT bundles that colocalised with fascin compared to non-fascin-associated bundles in the same migrating haemocyte, and combined this data from multiple different cells and embryos for analysis. Data showed a significant increase in the MT growth phase time in non-fascin-associated bundles (Fig. 1G; Movie 2), in agreement with analysis in fascin-depleted human cancer cells. Taken together, these data support a new and conserved role for fascin in the regulation of MT stability both *in vitro* and *in vivo*.

### Fascin associates directly with MTs *in vitro* and in cells

**Fig. 2.**
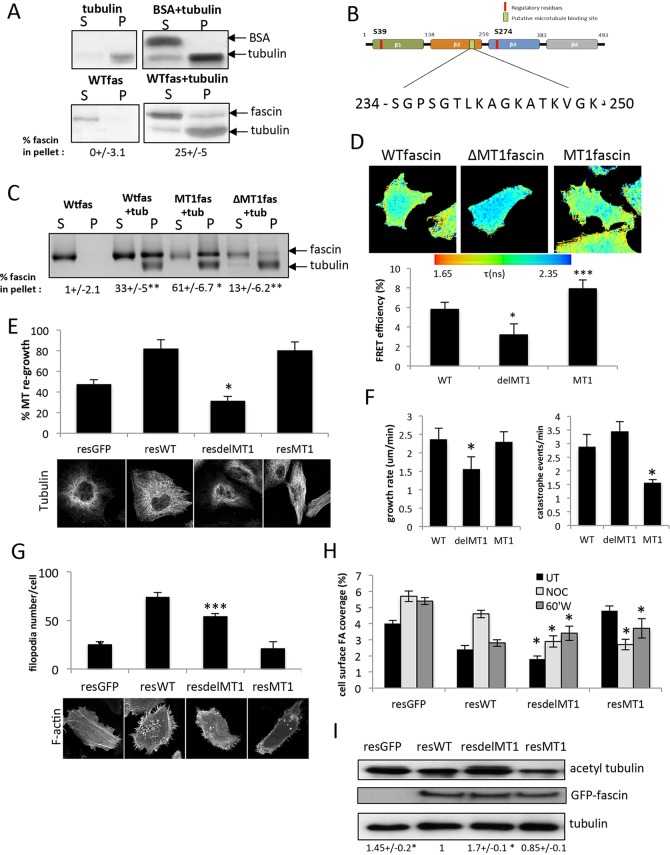
**Fascin binds to MTs.** (A) Example images of silver-stained gels showing levels of purified WT fascin (WTfas) and tubulin in the supernatant (S) or pellet (P) fractions following co-sedimentation. BSA was used as a protein control (top panel). Values beneath show the mean±s.e.m. percentage of fascin in the pellet from three independent experiments. (B) Schematic showing identified putative MT1 binding domain in fascin. (C) Co-sedimentation analysis of purified WT versus mutant fascins with tubulin (tub). MT1fas denotes mutated fascin, ΔMT1fas has a deleted MT1 domain. Values beneath show the mean±s.e.m. percentage of fascin in the pellet from four independent experiments. (D) Example fluorescence lifetime maps of fascinKD cells expressing GFP-tagged WT, ΔMT1- or MT1-fascin and tubulin–mCherry. A pseudocolour lifetime scale is shown where warmer colours denote low lifetimes and therefore high FRET. The graph beneath shows quantification of FRET efficiency for each condition from 18 cells per condition over three independent experiments. Mean±s.e.m. values are shown. (E) Quantification of MT re-growth in HeLa fascinKD cells re-expressing GFP only (resGFP) or GFP-tagged WT, ΔMT1- or MT1-fascin (resWT, resdelMT1 and resMT1, respectively) at 60 min after NOC washout. Fixed cells stained with anti-tubulin antibodies were scored as in Fig. 1. Example images are shown below the graph. (F) Analysis of MT growth rate (left graph) and catastrophe events/min (right graph) in fascinKD HeLa cells re-expressing GFP-tagged WT, ΔMT1- or MT1-fascin. Mean±s.e.m. are calculated from 25 MTs per cell in five cells per experiment and three independent experiments. Example movies are shown in Movie 3. (G) Quantification of filopodia number/cell in MDA MB 231 fascinKD cells re-expressing GFP alone, or GFP-tagged WTfascin or the MT1 or ΔMT1 mutants. Example images are shown below the graph. (H) Quantification of the percentage focal adhesion surface area coverage in FascinKD cells expressing WT or MT1 mutant GFP–fascin that were either untreated (UT), treated with NOC for 20 min (NOC) or at 60 min post-NOC washout (60′W). *n*≥35 cells quantified across three independent experiments. Mean values±s.e.m. are shown. (I) Western blots of acetylated tubulin in HeLa fascin knockdown cells expressing GFP or the specified fascin mutants. Blots were re-probed for GFP and tubulin. Numbers below blots are means±s.e.m. from three experiments. *P<0.05; **P<0.01; ***P<0.001 compared to controls (one-way ANOVA).

One possible explanation for the observed fascin-dependent defects in MT dynamics is a direct or indirect association of fascin with the MT network. To first explore the possibility of a direct interaction, we performed *in vitro* co-sedimentation assays between *in-vitro*-purified fascin, and polymerised and taxol-stabilised purified tubulin. In the absence of tubulin, fascin was not detectable in the high-speed pellet; however, ∼25% of fascin, but not a bovine serum albumin (BSA) protein control, was observed to co-sediment in the high-speed fraction when combined with Taxol-stabilised MTs (Fig. 2A), suggesting that fascin and MT might directly interact. We next sought to identify a potential binding domain for MTs within the sequence of fascin. Although MT-binding domains are diverse in sequence and charge, recent bioinformatics approaches have proposed some common features in these domains across different MT binding protein classes (Cao and Mao, 2009). Inspection of the fascin protein sequence revealed a basic, lysine-rich putative MT-binding sequence within the C-terminal region of the second β-trefoil domain (amino acids 234–250; Fig. 2B). To further investigate whether this domain might play a role in fascin–MT binding, we generated purified fascin protein mutated at five residues within this region (S/S/T/K/K>A; herein referred to as MT1) or lacking this 16-amino-acid stretch (herein referred to as ΔMT1) and performed MT co-sedimentation assay analysis as above. Previous studies have demonstrated that mutating regions within MT-binding domains to alanine residues in other MT-binding proteins, such as kinesin and doublecortin, results in more stable association (Klumpp et al., 2003; Makrantoni et al., 2014; Schaar et al., 2004; Zhu et al., 2010). Consistent with these studies, purified mutant MT1-fascin showed significantly higher association with polymerised MTs compared to wild-type (WT) fascin protein, whereas ΔMT1-fascin association with the MT pellet was significantly lower (Fig. 2C). These data combined demonstrate that fascin can directly bind to polymerised MTs *in vitro*, and that amino acids 234–250 might act as a stabilising interface in this association.

To confirm that the direct interaction between fascin and MTs also occurred in the context of an intact cell, we performed fluorescence lifetime imaging microscopy (FLIM) to analyse fluorescence resonance energy transfer (FRET) between GFP–fascin and tubulin–mCherry [as we have previously for fascin–F-actin binding (Jayo et al., 2012; Zanet et al., 2012)]. Fluorescence lifetime maps of individual cells and cumulative FRET efficiency analysis both revealed a direct association between WT fascin and MTs (Fig. 2D). In agreement with our *in vitro* data, ΔMT1-fascin showed significantly reduced FRET with tubulin in cells, whereas MT1-fascin showed significantly higher association with MTs under the same conditions (Fig. 2D). Tubulin–GFP and mCherry–WTfascin exhibited similar levels of FRET, whereas GFP-fascin co-expressed with mCherry alone showed no FRET, demonstrating that binding was not non-specific or dependent upon the fluorophore pairs used (data not shown). Phenotypic analysis revealed that MT re-growth following NOC washout occurred efficiently in fascin-depleted cells re-expressing GFP-tagged WT or MT1-fascin, but was significantly delayed in ΔMT1-fascin-expressing cells, again supporting a role for fascin binding to tubulin in controlling MT dynamics (Fig. 2E). Moreover, analysis of MT dynamics in cells co-expressing WT, ΔMT1- or MT1-fascin–GFP and tubulin–mCherry revealed a reduced MT growth rate in ΔMT1-fascin cells and a significant reduction in catastrophe events in cells expressing MT1-fascin (Fig. 2F; Movie 3). Thus we conclude that fascin and MTs are able to form a direct complex both *in vitro* and *in vivo* and that this association plays a role in controlling MT dynamics.

### Fascin–MT binding occurs independently of fascin–actin binding

To determine whether the fascin region 234–250 also contributed to fascin–actin binding, purified MT1- and ΔMT1-fascin were assessed for their ability to bundle fluorescent F-actin using co-sedimentation assays followed by confocal microscopy. Images of *in vitro* polymerised Alexa-Fluor-488-labelled F-actin showed that MT1-fascin was unable to support bundling to levels seen with the WT fascin protein; however, F-actin bundles were still visible in the preparation containing ΔMT1 (Fig. S2B). Similarly, when the same assay was performed and analysed biochemically, ΔMT1-fascin and WT fascin were able to co-sediment with F-actin to a similar degree *in vitro*, whereas MT1-fascin showed a significant reduction in pelleting (Fig. S2C). Moreover, FLIM analysis of FRET between fascin and F-actin demonstrated a similar association between ΔMT1-fascin and F-actin compared to WT fascin (Fig. S2D), further supporting our *in vitro* evidence that amino acids 234–250 in fascin are not required for F-actin binding. These data demonstrate that disrupting fascin–MT binding does not interfere with fascin–F-actin binding *in vitro*. To further determine whether fascin MT1 or ΔMT1 mutants altered cytoskeletal assembly in cells, we expressed GFP-tagged forms of fascin mutants in fascinKD cells and analysed F-actin organisation compared to control cells. In agreement with the *in vitro* analysis, fascinKD cells re-expressing MT1-fascin, which binds more stably to MTs, did not exhibit restored filopodia formation compared to WT fascin rescued cells (Fig. 2G). However, a partial rescue of filopodia assembly was seen in cells expressing ΔMT1-fascin compared to fascinKD cells, demonstrating that non-MT binding mutants of fascin are still able to support F-actin bundling (Fig. 2G). The partial rescue of filopodia assembly in the cells expressing the non-MT-associated shows that this form of fascin is still functional and able to position at the cell periphery in order to associate with and bundle F-actin. Taken together, these data demonstrate that fascin–actin bundling can occur when fascin–MT binding is disrupted, suggesting the two events are independent of one another and potentially mutually exclusive.

### Fascin-dependent focal adhesion assembly requires fascin–MT binding

We next asked whether the direct interaction between fascin and MTs functionally contributed to adhesion dynamics. To this end, adhesion disassembly following NOC washout was quantified in fascinKD cells re-expressing WT, MT1 or ΔMT1 fascin tagged to GFP as in Fig. 1. Cells expressing the MT1- or ΔMT1-fascin mutants were both unable to rescue adhesion dynamics following NOC washout to levels seen in WTfascin-expressing cells (Fig. 2H). ΔMT1-fascin cells failed to disassemble adhesions following NOC washout, in agreement with the lack of MT recovery in these cells at the same time point (Fig. 2E). Surprisingly however, MT1-fascin-expressing cells showed a significant increase in adhesion size following NOC washout (Fig. 2H) suggesting that stabilising the fascin–MT complex also results in more stable focal adhesions. We postulated that the reduced focal adhesion dynamics in these cells might correlate with altered MT stability. In support of this, analysis of acetylated tubulin levels by western blotting under basal conditions demonstrated higher levels in ΔMT1-fascin-expressing cells and less stable MTs in MT1-fascin cells in both HeLa and MDA MB 231 cells (Fig. 2I and data not shown). These data demonstrate that fascin–MT binding plays a role in MT stability and that this contributes to focal adhesion disassembly.

### Fascin-dependent cell migration requires both MT and F-actin binding

**Fig. 3.**
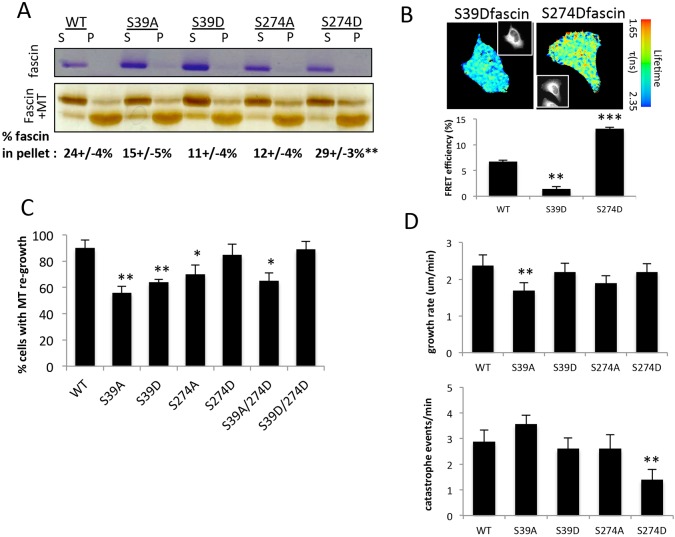
**Phosphorylation of fascin regulates MT and adhesion dynamics.** (A) Example images of Coomassie and silver-stained gels from co-sedimentation analysis of purified WT versus mutant fascin alone (top gel) or with tubulin (bottom gel) in the supernatant (S) or pellet (P) fractions. Values beneath show the mean±s.e.m. percentage of fascin in the pellet from three independent experiments. (B) Example fluorescence lifetime maps of fascinKD cells expressing S39D- or S274D-fascin–GFP and tubulin–mCherry (shown in inset panels). A pseudocolour lifetime scale is shown next to each image. Graph shows quantification of FRET efficiency for WT, S39D- and S274D-fascin-expressing cells from 15 cells per condition over two independent experiments. Mean±s.e.m. values are shown. (C) Quantification of MT re-growth from images of fixed MDA MB 231 FascinKD cells expressing WT or mutant GFP–fascin at 60 min post-NOC washout. *n*=30 cells were quantified across three independent experiments. (D) Analysis of MT growth rate (left graph) and catastrophe events/min (right graph) in fascinKD HeLa cells re-expressing WT or mutant GFP–fascin variants as specified. Values are calculated from 25 MT per cell in five cells per experiment over three independent experiments. Example movies are shown in Movie 4. *P<0.05; **P<0.01; ***P<0.001 compared to controls (one-way ANOVA).

Our data have demonstrated that fascin can associate with MTs directly, and that binding to MTs and F-actin might occur in a mutually exclusive manner. We therefore next asked whether the known sites in fascin that control actin binding might also contribute to the formation of the fascin–MT complex. To determine this, we repeated the MT co-sedimentation assays with purified WT fascin or the previously characterised mutants of fascin to mimic phosphorylated (S>D) or non-phosphorylated (S>A) states at S39 or S274 (Zanet et al., 2012). S39D, S274A and S274D are all unable to bundle F-actin as efficiently as WT fascin, whereas S39A fascin forms highly stable F-actin bundles *in vitro* and in cells (Ono et al., 1997; Vignjevic et al., 2006; Zanet et al., 2012). All fascin mutant proteins showed a degree of co-sedimentation with MTs (Fig. 3A); however, S274D-fascin showed significantly higher binding compared to WT protein and other mutants (Fig. 3A), as was also observed for the MT1-fascin mutant (Fig. 2C). To further confirm that S274D-fascin was more strongly associated with MT in cells, we performed FRET-FLIM analysis between fascin and tubulin in cells as described in Fig. 2D. FRET efficiency data from multiple cells demonstrated a direct interaction between S274D-fascin–GFP and tubulin–mCherry that was significantly higher than both WT fascin and S39D-fascin (Fig. 3B). This data again supports the notion that F-actin-binding mutants of fascin do not also lead to impaired fascin–MT association, suggesting the two cytoskeletal binding events can be independently controlled.

We next tested whether the increased binding observed between S274D-fascin and MTs could impact on MT dynamics in cells. To determine whether actin-binding mutants of fascin could regulate MT growth in cells, we re-expressed GFP-tagged WT or mutant fascin proteins into MDA MB 231 cells depleted of endogenous fascin. Analysis of MT re-growth following NOC washout revealed that, of the four single-site mutants tested, only S274D-fascin was able to support MT network re-growth in cells as efficiently as WT fascin (Fig. 3C). To allow us to analyse potential crosstalk or redundancy between the two known actin-binding sites in this context, we generated double mutants (S39A/S274D and S39D/S274D) and re-expressed them in fascin-depleted cells. Both double mutants resulted in significantly impaired filopodia formation when expressed in fascin-depleted MDA MB 231 cells, demonstrating that disrupting contact between F-actin at S274 site was sufficient to suppress the F-actin-bundling activity (Fig. S3A). Interestingly, however, expression of S39D/S274D, but not S39A/S274D fascin supported efficient MT re-growth following NOC washout (Fig. 3C). This suggests that F-actin binding through the N-terminal-binding domain of fascin suppresses fascin-dependent MT assembly. This MT growth phenotype was further confirmed by live imaging of MT dynamics in fascinKD HeLa cells co-expressing GFP-tagged fascin variants and tubulin–mCherry. Quantification of movies and the resulting data demonstrated a significant reduction in MT growth rate in cells expressing S39A-fascin compared to other fascin forms, and a significant reduction in catastrophe events in S274D-fascin-expressing cells (Fig. 3D; Movie 4) in agreement with our analysis in fixed cells. Similarly, we also observed that expression of S289D-fascin in fascin-null *Drosophila* haemocytes (equivalent to S274D; Zanet et al., 2012) resulted in assembly of stable fascin–MT bundles and reduced MT dynamics *in vivo* compared to WT fascin (Movies 5 and 6). Collectively these data demonstrate that S274 within the C-terminal actin-binding site of fascin plays an important role in the regulation of fascin-dependent MT dynamics both *in vitro* and *in vivo*.

**Fig. 4.**
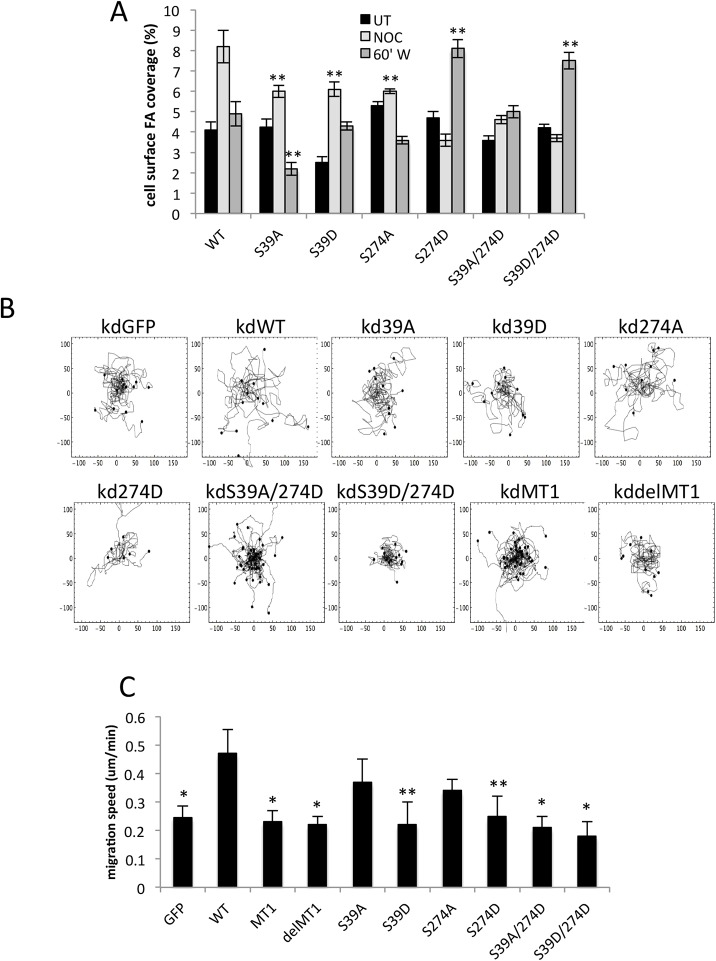
**Fascin–MT binding regulates cell adhesion dynamics and migration.** (A) Quantification of focal adhesion surface area coverage of MDA MB 231 FascinKD cells expressing WT or mutant GFP–fascin that were either untreated (UT), treated with NOC for 20 min (NOC) or at 60 min post-NOC washout (60′W). *n*=45 cells were quantified across three independent experiments. (B) Example migration tracks of MDA MB 231 FascinKD cells expressing WT or mutant GFP–fascin taken from time-lapse movies. (C) Quantification of migration speed as determined from tracks as shown in C. *n*=60 cells per condition. Mean±s.e.m. values are shown. *P<0.05; **P<0.01 compared to controls (one-way ANOVA).

To determine whether these MT-binding differences resulted in adhesion area changes, we quantified adhesion area in cells expressing S39A, S39D, S274A or S274D mutants of fascin. Data demonstrated that S39A, S39D and S274A mutants of fascin were able to support adhesion disassembly in response to NOC washout (Fig. 4A; Fig. S3B). Conversely, cells expressing S274D-fascin showed larger adhesions under both basal conditions and upon NOC washout (Fig. 4A), mimicking the effect seen in cells expressing the MT1-fascin mutant, which also similarly exhibits increased MT binding (Fig. 2). There were no changes in total adhesion protein levels in cells expressing fascin mutants (Fig. S3C). In agreement with the MT re-growth assay data, the adhesion disassembly defect in S274D cells was partially reversed in the S39A/S274D double mutant cells, but not in the S39D/S274D cells (Fig. 4A). These data together further suggest that S274 plays an important role in fascin–MT association and focal adhesion dynamics, potentially through disconnecting fascin from F-actin and promoting direct fascin­–MT binding.

In order to determine whether the defects in adhesion dynamics correlated with defective cell migration, we performed phase contrast time-lapse microscopy of fascinKD cells rescued with WT or mutant fascin proteins followed by single cell tracking and analysis. Quantitative comparisons of migration tracks demonstrated that cells expressing ΔMT1-fascin or S39D-fascin had slower migration speeds compared to cells rescued with WT protein (Fig. 4B,C), suggesting that loss of F-actin or MT binding is sufficient to impair fascin-dependent migration. Similarly, cells expressing MT1, S274D or S39A/S274D mutants of fascin that did not undergo efficient adhesion disassembly also showed significantly reduced migration speeds (Fig. 4B,C). These experiments provide correlative evidence for the idea that co-ordinated control of phosphorylation at S39 and S274 residues is required for fascin-dependent MT assembly, adhesion disassembly and cell migration.

### Fascin regulates activation of FAK

**Fig. 5.**
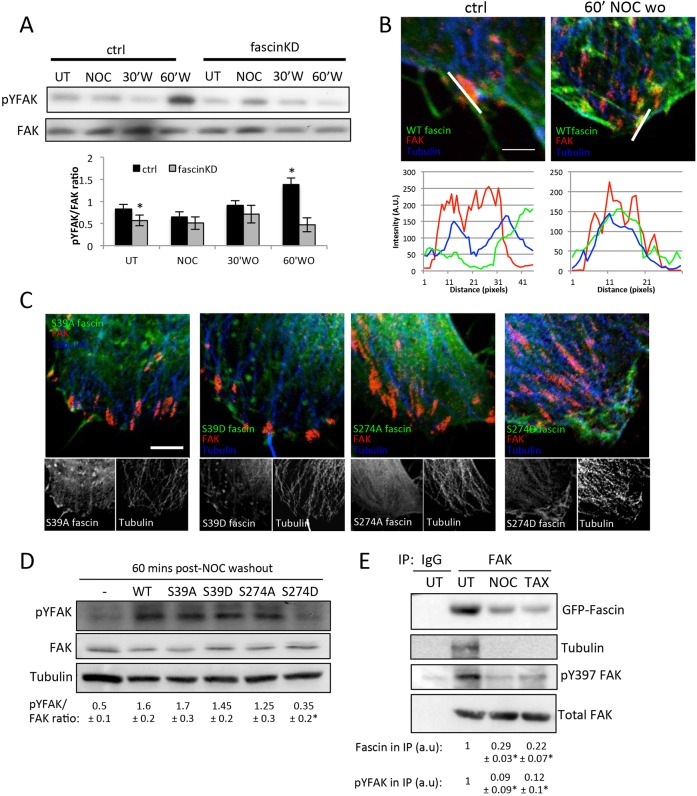
**Fascin regulates activation of FAK.** (A) Lysates from control (ctrl) and fascinKD MDA MB 231 cells that were either untreated (UT), treated with NOC for 20 min (NOC) or at 30 or 60 min post-NOC washout were subjected to western blot analysis for pY397-FAK (pYFAK) or total FAK. The graph beneath the blots shows densitometry quantification of pY397-FAK/total FAK levels from three independent experiments. Mean±s.e.m. values are shown. (B) Representative confocal images of regions of control MDA MB 231 cells that were untreated (UT) or following NOC washout (60′W) fixed and stained for endogenous fascin (green), FAK (red) and tubulin (blue). Merged images are shown. Scale bar: 2 μm. A profile of staining along the indicated line is shown underneath the images. (C) Example images of FAK staining (red) at focal adhesions at the periphery of fascinKD MDA MB 231 cells expressing the specified fascin–GFP constructs (green) co-stained for tubulin (blue). Merged images are shown in top panels, and fascin and tubulin channels are shown as black-and-white images below. Scale bar: 15 μm. (D) Western blot of lysates from fascinKD or the specified GFP–fascin rescued MDA MB 231 cells at 60 min post-NOC washout, probed for pYFAK, FAK or tubulin. Numbers beneath blots are a mean±s.e.m. densitometry quantification of pY397-FAK/total FAK levels from three independent experiments. (E) Western blots of lysates from control cells treated with NOC or Taxol and immunoprecipitated (IP) with control (IgG) or anti-FAK antibodies. Blots were probed for specified proteins. Relative fascin and pY397-FAK levels in each immunoprecipitation lane were quantified over four independent experiments (mean±s.e.m. densitometry values are denoted below each respective lane). *P<0.01 compared to control (A,D); *P<0.001 compared to untreated controls (E) (A,D, one-way ANOVA; E, Student's *t*-test).

We next asked how fascin–MT binding might mechanistically impact on focal adhesion signalling. FAK is a key component of focal adhesions and is known to recruit the non-receptor tyrosine kinase Src following auto-phosphorylation at Tyr397. The activation of FAK and induction of the FAK-Src complex has previously been shown to play a key role in adhesion dynamics through clathrin-dependent endocytosis of integrins and other adhesion components (Ezratty et al., 2009, 2005; Wang et al., 2011). Furthermore, MT-induced disassembly of adhesions requires activation of the FAK-Src pathway, and disruption of the FAK phosphorylation cycle leads to enhanced focal adhesion growth and stability (Hamadi et al., 2005; Wang et al., 2011). Thus, we postulated that fascin might operate upstream of the FAK–Src pathway to regulate adhesion dynamics. In order to determine whether fascin might regulate focal adhesion dynamics through this pathway, we quantified active FAK levels in lysates from control or fascinKD cells throughout the NOC washout assay. Data demonstrated that fascin-depleted cells exhibited significantly lower active FAK under basal conditions and also failed to activate FAK (through phosphorylation of Y397) at 60 min after drug washout and focal adhesion disassembly (Fig. 5A). Confocal imaging and linescan analysis of WT-fascin-expressing cells further revealed that a population of endogenous fascin colocalised with FAK and MTs at peripheral focal adhesion sites following NOC washout (Fig. 5B). Investigation of FAK and tubulin in cells expressing fascin mutants further revealed larger MT bundles overlapping with FAK-positive adhesions in S274D-fascin-expressing cells, and partial colocalisation between S39A-fascin and FAK, but not the other fascin mutants (Fig. 5C). Moreover, biochemical analysis of FAK phosphorylated at tyrosine (pY-FAK) levels following NOC washout in these cells demonstrated that re-expression of S274D-fascin did not support activation of FAK upon MT re-growth to the extent seen in cells expressing all other fascin forms (Fig. 5D). These data together suggest that fascin is required for activation of FAK during MT-induced adhesion disassembly, and that co-ordination between these functions likely requires a dynamic cycle between fascin–actin and fascin–MT binding.

Given the partial colocalisation between fascin, FAK and MTs we postulated that these proteins might be able to form a complex and that this complex might be dependent upon MT stability. To test this hypothesis, endogenous FAK was immunoprecipitated from fascinKD cells expressing WT-fascin–GFP under basal conditions or following treatment with NOC or Taxol to disrupt or stabilise MTs, respectively. Western blotting demonstrated that fascin was readily detectable in a complex with FAK and tubulin, and that this was reduced under both conditions that disrupted MT dynamics (Fig. 5E). Moreover, the FAK that was found in a complex with fascin and tubulin under basal conditions was phosphorylated at Y397, but this activation was significantly reduced in cells treated with NOC or Taxol (Fig. 5E). To further determine whether this complex was the result of a direct interaction between fascin and FAK, we performed pulldown experiments using purified fascin co-incubated with C-terminal or FERM domains of FAK tagged to GST. However, fascin did not bind to any FAK domain tested under these conditions, suggesting that these proteins do not directly interact *in vitro* (data not shown).

### Fascin–MT binding acts upstream of FAK and Src to regulate adhesion dynamics

**Fig. 6.**
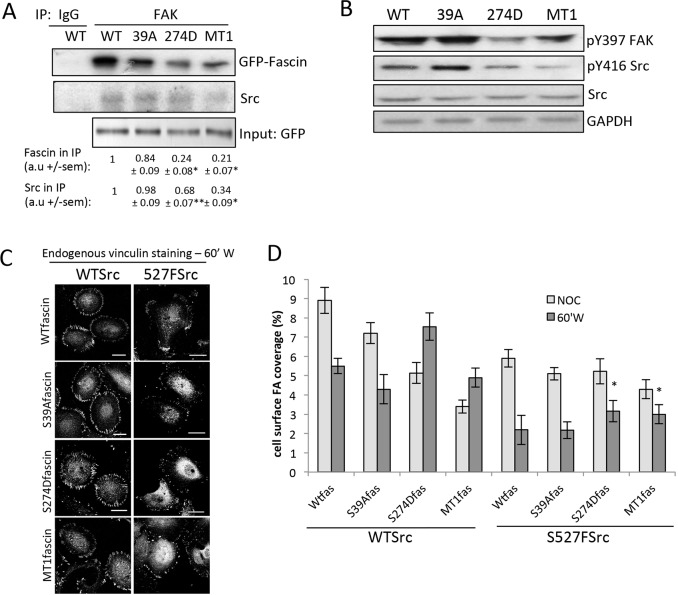
**Microtubule-dependent adhesion dynamics are regulated through a fascin–FAK–Src signalling pathway.** (A) Western blots of lysates from fascinKD cells expressing WT, S39A-, S274D- or MT1-fascin–GFP immunoprecipitated (IP) with control (IgG) or anti-FAK antibodies. Blots of immunoprecipitations (left panel) were re-probed for specified proteins. Values are denoted below each respective lane and represent relative intensity of each band as a mean±s.e.m. of the four experiments. (B) Western blots of lysates from fascinKD cells expressing GFP-tagged WT, S39A-, S274D- or MT1-fascin probed with the specified antibodies. (C) Representative images of vinculin staining in fascinKD HeLa cells expressing GFP-tagged WT Src (WTSrc) or constitutively active S527F Src (527FSrc) with WT, S39A or 274D-fascin–mFP at 60 min post-NOC washout. Scale bars: 20 μm. (D) The percentage focal adhesion coverage per cell was quantified from cells treated with NOC for 20 min (NOC) or at 60 min following NOC washout (60′W). *n*=45 cells were quantified across three independent experiments. Mean values±s.e.m. are shown. *P<0.05, **P<0.01, compared to untreated controls (A); *P<0.01 compared to WT fascin (D) (one-way ANOVA).

To determine whether FAK–fascin complex formation was regulated by fascin–MT binding, we performed FAK immunoprecipitations from fascinKD cells expressing WT, S39A, S274D or MT1 mutant forms of GFP–fascin. Data showed similar levels of WT and S39A-fascin in complex with FAK; however, the S274D and MT1-fascin mutants, that both exhibit increased MT binding, both showed significantly lower association in the complex (Fig. 6A). Moreover, re-probes of these immunoprecipitation complexes revealed the presence of Src, which also showed lower co-association with FAK in cells expressing S274D- or MT1-fascin (Fig. 6A). Taken together, these data suggest that fascin, FAK and Src are able to form a complex and that this is dependent upon both fascin–MT binding and the presence of a dynamic MT cytoskeleton.

As S274D-fascin resulted in much larger focal adhesion and lower active FAK, we hypothesised that S274D-fascin might prevent effective downstream signalling to Src. To address this, we first analysed basal levels of active FAK and Src in fascin-depleted cells re-expressing either GFP-tagged WT, S39A-, S274D- or MT1-fascin. Western blots showed no change in total levels of FAK or Src in any of the cells expressing mutant fascins (Figs 6B and 5D). However, decreased activity of both FAK and Src were seen in S274D- and MT1-fascin-expressing cell lysates (Fig. 6B), suggesting that fascin–MT binding affects activation of these key adhesion molecules and in agreement with similar findings in NOC washout conditions (Fig. 5D). We next wanted to determine whether Src was a key molecule downstream of fascin–MT binding in controlling focal adhesion dynamics. To determine this, we expressed WT or constitutively active Src(Y527F)–GFP in fascinKD cells rescued with WT, S39A, S274D or MT1 fascin tagged to mRFP, and analysed adhesion disassembly following NOC washout. In the presence of expressed WT Src–GFP, NOC washout resulted in adhesion disassembly in WT- and S39A-fascin-expressing cells, but not those rescued with S274D- or MT1-fascin mutants (Fig. 6C,D), similar to the response seen previously in control cells. However, co-expression of Src(Y527F) resulted in restoration of adhesion disassembly to comparable levels in all cells (Fig. 6C,D), suggesting that constitutive activation of Src is able to override the adhesion stabilising effect of S274D- and MT1-fascin mutants. Taken together, these findings demonstrate that the association of fascin with MTs, FAK and Src promotes activation of the FAK–Src complex and facilitates efficient MT-dependent focal adhesion disassembly.

## DISCUSSION

**Fig. 7.**
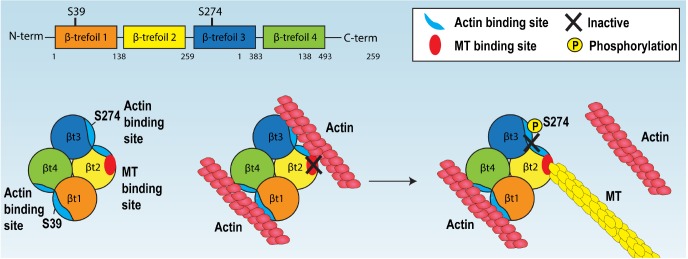
**Proposed model of fascin cytoskeletal associations.** Model for fascin-dependent association with F-actin or MTs. See Discussion for details.

Fascin has been well characterised as an actin-bundling protein that can regulate cell migration as well being an emerging important prognostic marker for metastatic disease (Bi et al., 2012; Gao et al., 2012; Hashimoto et al., 2006; Jayo and Parsons, 2010; Li et al., 2008). Here, we demonstrate for the first time that fascin can associate directly with MTs. We show that fascin–MT binding is in part controlled through a binding site within the second β-trefoil domain, and that disrupting the interaction between fascin and MTs does not significantly impact on fascin F-actin binding. We further show that S274 is a key residue for fascin-mediated regulation of MT binding and that a phospho-mimicking mutation of this residue results in highly stable MT binding. Disruption of the fascin–MT complex leads to reduced focal adhesion disassembly and impaired cell migration through control of FAK–Src signalling. Our data support a model whereby fascin–actin bundling and fascin–MT binding act in synergy to control focal adhesion dynamics and cell motility (Fig. 7). We propose that phosphorylation of S274 can potentially disrupt the interface between F-actin and the actin-bundling site located between β-trefoils-2 and -3 in fascin, thus revealing the MT1 binding site located on the adjacent β-trefoil 2 domain and promoting the switch from F-actin to MT binding (Fig. 7). The available crystal structures of fascin (Jansen et al., 2011) show that the MT1 binding site we have identified is exposed within the molecule, further supporting our hypothesis that F-actin bundling provides a potential mask across this domain to prevent MT engagement with fascin. Our data also provides new regions of fascin to explore for rational design for agents that target fascin–actin and fascin–MT binding and potentially target cancer cell metastasis.

MTs have previously been shown to promote adhesion disassembly, through delivery of disassembly or relaxation factor(s) and/or through activation of the Rho family GTPases (Ezratty et al., 2005; Palazzo et al., 2004; Waterman-Storer et al., 1999). Our data demonstrates that fascin can be found in a complex with FAK, and the complex formation is coincident with a transient re-localisation to peripheral adhesion sites. Moreover, fascin is required for normal MT dynamics and that loss of fascin leads to a more stable MT network. These observations strongly suggest that fascin can associate with distinct, specific binding partners within different subcellular sites depending on phosphorylation status at both N- and C-terminal sites. Our data would further support the concept that fascin that is phosphorylated at S274 preferentially locates at peripheral membrane sites. Our study additionally shows that expression of fascin mutants that bind to MT and suppress MT dynamics also result in reduced active FAK and Src levels at adhesion sites. Our finding that defective adhesion disassembly in cells expressing S274D- or MT1-fascin can be rescued by expression of constitutively active Src would suggest that fascin acts upstream of the FAK–Src complex. Interestingly, stable MT networks have also been proposed to transmit mechanical stress signals to activate Src at focal adhesions, providing a further potential explanation of how fascin might regulate this pathway (Na et al., 2008). Notably, we did not detect significant levels of fascin directly within focal adhesions by fluorescence confocal microscopy or TIRF microscopy in MDA MB 231 cells, HeLa cells or NIH 3T3 fibroblasts under basal conditions. In agreement with our findings, none of the previous studies have reported adhesion localisation of endogenous or overexpressed fascin in a wide range of normal and transformed cells (Anilkumar et al., 2003; Jansen et al., 2011; Ross et al., 2000; Schoumacher et al., 2014; Vignjevic et al., 2006; Yamakita et al., 1996; Yang et al., 2013). This wealth of data suggests that fascin is not a common component of focal adhesions; one exception being a recent study demonstrating fascin recruitment to adhesions in HCT116 cells perhaps due to specific differences in the motile or contractile behaviour of these cells (Elkhatib et al., 2014). However, we have shown that fascin can be transiently recruited to focal adhesions at low levels following NOC washout when cells are actively disassembling adhesions and MTs are highly dynamic. This model is further supported by a very recent proteomics study showing that fascin is one of a large family of proteins that are specifically enriched in complexes with active integrins (Byron et al., 2015). Thus it seems likely that fascin is transiently associated with populations of active adhesion molecules through a so-called ‘kiss-and-run’ mechanism and that this is controlled through its relative affinities for F-actin and MT cytoskeletons.

There are a number of mechanisms that might control local changes in fascin binding to different cytoskeletal networks during migration. We have recently shown that activation of the RhoA–ROCK–LIMK signalling pathway promotes fascin-mediated actin bundling through a direct association between fascin and LIMK within filopodia (Jayo et al., 2012). Control of MT stability by fascin has the potential to act as a feedback loop to regulate fascin function. Recent studies have shown that the RhoA exchange factor GEF-H1 (also known as ARHGEF2) is released from MTs and activated following MT destabilisation, leading to enhanced RhoA activity that in turn promotes invasion within dense 3D collagen matrices (Chang et al., 2008; Heck et al., 2012). Local activation of RhoA following MT destabilisation might therefore favour a switch from fascin–MT to fascin–actin association and thus promote actin assembly and membrane protrusion. Recent reports have shown that a dynamic MT network is required for membrane associated PKC activation and active PKC then promotes association of EB1 (also known as MAPRE1) and KIF17 with growing MT plus tips (Espenel et al., 2013; Nakhost et al., 2002; Schober et al., 2012). As PKC is known to act on fascin to promote a non-actin bundling conformation through phosphorylation on S39, it is possible that an active pool of PKC proximal to MT plus tips would also potentially favour local fascin disconnection from F-actin bundles.

Fascin has previously been shown to promote the migration and invasion of cancer cells, the assumption being that this was mediated entirely through fascin-actin dependent control of filopodia and invadopodia structures (Hashimoto et al., 2007; Li et al., 2010; Schoumacher et al., 2010). Our current data suggests that fascin might also contribute to these processes through regulating MT dynamics and adhesion stability. Previous studies have shown that stable MTs can be concentrated towards the leading edge of polarised cells or within matrix-degrading adhesions, and that this contributes to directed cell migration and chemotaxis (Diaz-Valencia et al., 2011; Montagnac et al., 2013; Schoumacher et al., 2010; Wu et al., 2008). Each cytoskeletal network plays a distinct role in cellular architecture, plasticity and responses to a changing extracellular environment, but the way in which this is co-ordinated remains poorly understood. Given that fascin is now a widely recognised prognostic marker of metastatic disease, it will be important to investigate whether strategies targeted towards dual inhibition or modulation of fascin–actin and fascin–MT binding can slow or restrict invasive disease.

## MATERIALS AND METHODS

### Reagents and antibodies

Antibodies were purchased from the following suppliers: mouse anti-β-tubulin, mouse anti-acetylated tubulin, mouse anti-phosphorylated tyrosine (pY) and mouse anti-vinculin antibodies were all from Sigma-Aldrich; rabbit anti-α/β tubulin and mouse anti-Src were from Cell Signaling Technology; mouse anti-fascin and anti-mouse-IgG or rabbit-IgG secondary antibodies conjugated to horseradish peroxidase (HRP) were from Dako; mouse anti-FAK antibody was from Santa Cruz Biotechnology; antibody against FAK phosphorylated at Y397 and all Alexa-Fluor-dye-conjugated secondary antibodies and Phalloidin were from Invitrogen. Nocodazole, taxol and cytochalasin D were obtained from Sigma-Aldrich.

### Cell culture and transfection

MDA-MB-231 human breast carcinoma, 293T and HeLa cervical cancer cells were maintained at 37°C in Dulbecco's modified Eagle's medium (DMEM; Sigma-Aldrich) with 4500 mg glucose/l supplemented with 10% fetal bovine serum (FBS; Sera Laboratories), 1% L-Glutamine and 1% penicillin-streptomycin (both from Gibco). NIH3T3 cells were maintained in DMEM with 10% newborn calf serum (NCS). Cells were transfected in Optimem serum free medium (Gibco) using Lipofectime transfection reagent according to manufacturer's instructions (Invitrogen Life Technologies).

### Fly stocks

We used the following lines obtained from the Bloomington *Drosophila* Stock Center and the community: *sn*^28^, upstream activation sequence (UAS)-GFP-Fascin (Zanet et al., 2009), UAS-mCherry-Fascin (Zanet et al., 2012), UAS-GFP-Clip (MT-binding domain of human Clip-170) and UAS-Spastin-GFP (Stramer et al., 2010).

### Plasmids, cloning and virus production

Human lentiviral short hairpin RNA (shRNA) targeting fascin was cloned into the pLentiLox 3.7 (LL3.7), kindly donated by Jo Adams (University of Bristol, UK). The sequence of the shRNA (a gift from James Monypenny, King's College London, UK) used to knockdown fascin was 5′-GCCTGAAGAAGAAGCAGAT-3′ directed to the first exon of the human fascin gene, isoform 1 (*FSCN1*). Additionally, a scrambled sequence of the shRNA was cloned into the LL3.7 vector and used as a control. Scrambled and fascin-targeting shRNA sequences were also cloned into a pLKO lentiviral vector (RNAi Consortium) between the *Age*I and *Eco*RI sites. shRNA-resistant human fascin cDNA in which the shRNA target sequence was changed to 5′-GCCTGAAAAAAAAACAGAT-3′ (underlined letter indicate changes) and its wild-type and mutant forms were generated and tagged in the N-terminal site with pEGFP (Clontech). The four single fascin mutant forms (S39 and S274) were mutated to either a non-phosphorylatable alanine (A) or a phospho-mimic aspartic acid (D) and were generated as described previously (Zanet et al., 2012). Double mutant fascins were generated through mutation of the S274 in the S39 mutant background. For recombinant fascin purification, the cDNA encoding wild-type human fascin and its mutants were cloned between the *Not*I and *Xho*I restriction sites in the p-ET30a(+) vector (Novagen, UK). Fascin MT1 and ΔMT1 mutants were generated on the pEGFP-fascin and p-ET30a-fascin backbones by site-directed mutagenesis using the following oligonucleotides: 5′-CCCGCCGGCGCGCTCGCGGCGGGCGCGGCCACCAAGGTGGGC-3′ and 5′-CCCGCCTTGAGCGCGCCGGCGGGCCCCGCCGGCGCCAGGTAAC-3′ for MT1, and 5′-CGTTACCTGGCGCCGGACGAGCTCTTTGCTC-3′ and 5′-GAGCAAAGAGCTCGTCCGGCGCCAGGTAACG-3′ for ΔMT1. Vinculin–mRFP was as described previously (Worth et al., 2010). The human α-tubulin cDNA was cloned in mCherry-tagged lentiviral vector pLVX (a gift from James Monypenny) between the *Xho*I and *Bam*HI-HF sites. The following primers were used for PCR: 5′-CTCGAGGGAGGTGGAATGCGTGAGTGCATCTCCAT-3′ and 5′-GGATCCTTAGTATTCCTCTCCTCTT-3′. The cDNAs encoding the GST-tagged FERM domain, kinase and C-terminal domain of FAK were generated by Margaret Frame (University of Edinburgh, UK). Lentivirus was produced and cells infected as described previously (Scales et al., 2013).

### Protein purification and pulldowns

BL21 cells containing DNA of interest were grown overnight at 37°C. The culture was diluted (1:100) with fresh broth and cells grown at 37°C in a shaking incubator for 3–4 h. Protein production was induced with isopropyl β-D-1-thiogalactopyranoside (IPTG, 0.2 mM) and incubated for a further 3–4 h. The bacterial culture was pelleted by centrifuging at 1820 ***g*** for 15 min, 4°C. The pellet was then resuspended in 50 µl ice-cold PBS per ml of original culture volume, containing protease inhibitors (Calbiochem) and disrupted by sonication at 10 A (with a Q800 instrument, Fisher Scientific, UK) for ten times of duration 10 s with a pause of 10 s between (on ice). The solution was centrifuged at 1820 ***g*** for 30 min at 4°C and the supernatant collected. Ni-NTA agarose beads (Qiagen, UK) were packed on chromatography disposable columns (Thermo Scientific) and washed with lysis buffer (50 mM sodium phosphate, 10 mM imidazole and 300 mM sodium chloride, pH 8]. Subsequently, beads were added to the supernatant at 5 µl beads per 1 ml original culture and left to mix overnight at 4°C. The beads were spun down and washed with buffers containing increasing concentration of imidazole. A high concentration of imidazole was used to elute the proteins. The eluted protein was dialysed against storage buffer (1× PBS, 10 mM imidazole and 10% glycerol, pH 8) for at least 2 h at 4°C. Proteins were recovered and used for further experiments. Stocks of protein were kept at −80°C and SDS-PAGE was run to check protein purification. Silver or Coomassie staining was used to detect binding in MT and actin co-sedimentation experiments.

### Western blotting

Samples for western blotting were prepared from cell lysates, pulldown and/or immunoprecipitation experiments. The protein samples were run on SDS-PAGE gels and membranes probed with the primary antibody overnight at 4°C or for 3 h at room temperature. The membranes were then washed three times for 10 min each with PBS Tween (0.1% Tween in PBS) prior to incubation with HRP-conjugated secondary antibodies for 1 h at room temperature. Membranes were then washed a further three times and proteins were detected with the ECL chemiluminescence kit (Thermo Scientific, UK) on a Bio-Rad imager. For reprobing, blots were stripped with Re-blot strong (10×) (Chemicon) diluted to 1× in distilled water for 10 min at room temperature, and then blocked and incubated with antibodies as above.

### Immunoprecipitation

Control or fascin knockdown MDA-MB-231 cells expressing GFP-tagged WT or mutant fascin were lysed in 250 μl 50 mM Tris-HCl (pH 7.2), 150 mM NaCl, 1 mM EDTA, 1% Triton X-100, protease inhibitor cocktail, phosphatase inhibitors, 50 mM sodium fluoride and 1 µM calyculin on ice. Lysates were pre-cleared with 50 μl washed A/G agarose affinity matrix suspension rotating at 4°C for 30 min, and 50 μl of lysate was kept. A/G agarose affinity matrix suspension was washed three times with cold PBS prior to use. Antibody or control IgG was pre-incubated with beads for 3 h rotating at 4°C. Then, excess antibody was removed by washing three times with PBS, and pre-cleared lysate was added to each antibody A/G agarose affinity matrix suspension mix overnight rotating at 4°C. A/G agarose affinity matrix suspension was washed three times for 10 min in immunoprecipitation buffer. Lysates were boiled at 95°C and centrifuged to clear cell debris before use. 40 μl of each sample was loaded in each well of 8% or 10% SDS-PAGE gels and subjected to SDS-PAGE, followed by western blotting. Each immunoprecipitation was performed at least three times and quantified using densitometry analysis.

### MT and actin sedimentation analysis

For MT analysis, bovine brain tubulin (Cytoskeleton) was diluted in general tubulin buffer (GTB; 50 mM PIPES pH 6.8, 1 mM EGTA and 1 mM magnesium chloride) at a final concentration of 10 µg/µl. 5 µg of purified tubulin was polymerised for 15 min at 37°C in tubulin polymerisation buffer (GTB, 10% glycerol, 5 mM magnesium chloride, and 1 mM GTP). Increasing concentrations of Taxol (Sigma) were added every 10 min. MT structure was confirmed by electron microscopy. The mixture was then layered onto 60 µl of tubulin-pelleting buffer (GTB, 50% glycerol, 1 mM GTP and 80 μM Taxol) and centrifuged at 20,000 ***g*** for 20 min at 25°C. The buffer was prepared in 50% glycerol, which allows polymerised tubulin (MT) sedimentation. The MT pellet was resuspended in 60 µl of GTB plus 1 mM GTP, 80 μM Taxol. Polymerised tubulin corresponding to ∼5 µg (1 µmol) was incubated with His_6_-tagged WT or mutant fascin protein (1:1 ratio) in GTB for 30 min at 25°C. Each reaction mixture was then layered onto 20 µl of GTB and reactions were centrifuged at 20,000 ***g*** for 30 min at 25°C. As a control, MT, WT and mutant fascin proteins were subjected to centrifugation alone in every co-sedimentation experiment. 5× loading buffer was added to supernatants (unbound fraction), and each pellet was resuspended in 40 µl of GTB (with 15% glycerol added) and 2× loading buffer was added. The supernatant (S) and pellet (P) fractions were recovered by SDS-PAGE and stained with silver staining. Protein bands were quantified by densitometry. F-actin co-sedimentation experiments with fascin were carried out as previously described (Zanet et al., 2012). Protein bands were quantified by densitometry.

### Confocal microscopy and image analysis

For NOC washout assays, cells were treated with NOC or DMSO (control) (Sigma-Aldrich) at 10 µM diluted in DMEM with 10% FBS for 15 min to completely disassemble MTs. After treatment, cells were either fixed and stained immediately or the NOC was washed out and replaced with fresh DMEM with 10% FBS for 30 or 60 min to allow MT re-growth followed by fixation with PFA. The cells were stained with antibodies against total tubulin or phospho-tyrosine (pY) or vinculin and imaged with confocal microscopy. For live-cell imaging of focal adhesion dynamics, specified cells expressing vinculin–mRFP were plated into glass-bottomed imaging chambers (Ibidi) in Phenol-Red-free medium containing NOC and subjected to time-lapse confocal microscopy before and after NOC washout. Resulting NIS Elements ND2 files were exported and used for analysis in ImageJ to determine the focal adhesion area and cell area as for fixed samples. Images of cells stained for total tubulin were scored for percentage of cells with a normal MT network (MT score). Images were thresholded in ImageJ and binarised to define the MT network followed by overlay of the cell boundary using phalloidin co-staining to define cell edges. Cells exhibiting polymerised MT bundles that were distributed throughout the cell body to the entire periphery of the cell with a visible MT organising centre (MTOC) were scored as 100% re-growth. Spreading cells exhibiting an MT network that was incompletely re-grown were scored according to the MT coverage as a function of cell area (between 95 and 5%); cells exhibiting a largely depolymerised or diffuse MT network or with only a visible MTOC present scored as 5% or below. MT re-growth was calculated as a function of cell area, thus controlling for smaller spread area detected in fascin-depleted cells. Quantification of cell size and focal adhesion size and number was performed by application of a threshold to all images using ImageJ to isolate and identify focal adhesions between 1 and 5 µm in size. Focal adhesion size data was normalised to the total cell area and presented as the percentage of the cell occupied by focal adhesion. In all the analysis, at least 40 cells were evaluated in three different experiments (*n* numbers for each are defined in associated figure legends). Quantification of filopodia number was performed on confocal images of live cells co-expressing GFP–fascin (WT and mutants) and lifeact–mRFP. Filopodia were defined as membrane projections protruding more than 2 μm from the plasma membrane and containing F-actin and fascin. Images were acquired from multiple live cells, and filopodia number and length per cell were quantified from these images using ImageJ and averaged across multiple cells and experiments.

### FRET and FLIM

Fluorescence resonance energy transfer (FRET) was measured using fluorescence lifetime imaging microscopy (FLIM) as previously described (Parsons et al., 2008). Histogram data are plotted as mean FRET efficiency per cell, pooled from specified numbers of cells and experiments as detailed in figure legends. ANOVA was used to test statistical significance between different populations of data. Lifetime images of example cells are presented using a pseudocolour scale, whereby blue depicts normal GFP lifetime (no FRET) and red depicts lower GFP lifetime (areas of FRET where proteins are within <10 nm proximity).

### Live imaging of *Drosophila* embryos

UAS transgenic constructs encoding fluorescent proteins were expressed specifically in macrophages using the *singed*-Gal4 driver line (Zanet et al., 2012). Live embryos were mounted as previously described (Wood et al., 2006). Briefly, stage 15 embryos were dechorionated in bleach and mounted under coverslips on hydrophobic Lumox dishes (Sarstedt, Germany) in Voltalef oil 10S. Embryos were then imaged with a spinning disk microscope (UltraVIEW VoX PerkinElmer) with a 63× N.A. 1.4 objective acquiring one image every 30 s. Measurements of the length of fascin bundles were performed using Volocity software. MT arms were defined as polarised bundles of MT that anticipate direction of migration as previously described previously (Stramer et al., 2010).

### Microtubule imaging and tracking

Time-lapse movies of HeLa cells expressing tubulin–RFP were acquired on an Olympus IX71 epifluorescent microscope equipped with an EMCCD camera (Ixon, Andor, Belfast) using a 60×1.42 N.A. oil objective. To track single MTs at the cell periphery, acquired movies were subjected to a bandpass filter (20:2 pixels) in ImageJ, background subtracted using a rolling ball radius of 15 pixels, and a 3D Gaussian blur filter was applied. Resulting movies were overlaid with the originals to avoid image-processing-derived artefacts and single MT length was measured over time from a defined starting point proximal to the cell periphery. Frequency of catastrophe, growth rate and time spent in growth phase were quantified.

### Statistical analysis

All statistical tests were performed using a Student's *t*-test (Excel) or ANOVA (Prism) as relevant for specified data sets. Data are expressed as means±s.e.m. Experimental and cell (where relevant) *n* numbers are detailed in associated figure legends. Significance was taken as *P*<0.001, 0.005 and 0.05 and significance values were assigned in specific figures and/or experiments as detailed in individual figure legends.
